# Microfluidic and Lab-on-a-Chip Systems for Cutaneous Wound Healing Studies

**DOI:** 10.3390/pharmaceutics13060793

**Published:** 2021-05-26

**Authors:** Ghazal Shabestani Monfared, Peter Ertl, Mario Rothbauer

**Affiliations:** 1 Institute of Applied Synthetic Chemistry, Faculty of Technical Chemistry, Vienna University of Technology, 1060 Vienna, Austria; ghazal.shabestanimonfared@tuwien.ac.at; 2 Karl Chiari Lab for Orthopaedic Biology, Department of Orthopedics and Trauma Surgery, Medical University of Vienna, 1090 Vienna, Austria

**Keywords:** cell migration, cutaneous wound healing, wound healing assay, lab-on-a-chip, microfluidics, skin, microvasculature

## Abstract

Cutaneous wound healing is a complex, multi-stage process involving direct and indirect cell communication events with the aim of efficiently restoring the barrier function of the skin. One key aspect in cutaneous wound healing is associated with cell movement and migration into the physically, chemically, and biologically injured area, resulting in wound closure. Understanding the conditions under which cell migration is impaired and elucidating the cellular and molecular mechanisms that improve healing dynamics are therefore crucial in devising novel therapeutic strategies to elevate patient suffering, reduce scaring, and eliminate chronic wounds. Following the global trend towards the automation, miniaturization, and integration of cell-based assays into microphysiological systems, conventional wound healing assays such as the scratch assay and cell exclusion assay have recently been translated and improved using microfluidics and lab-on-a-chip technologies. These miniaturized cell analysis systems allow for precise spatial and temporal control over a range of dynamic microenvironmental factors including shear stress, biochemical and oxygen gradients to create more reliable in vitro models that resemble the in vivo microenvironment of a wound more closely on a molecular, cellular, and tissue level. The current review provides (a) an overview on the main molecular and cellular processes that take place during wound healing, (b) a brief introduction into conventional in vitro wound healing assays, and (c) a perspective on future cutaneous and vascular wound healing research using microfluidic technology.

## 1. Introduction

Mechanical injuries, burns, and illnesses are, among others, the leading causes of external or internal tissue damage or lesion, generally referred to as wounds. Breaks in the epithelial barrier, known as the cutaneous wound, disturb the skin’s primary function, which is the protection of the body against the external environment [1]. Following wounding and blood flow restriction (clotting), the injured tissue undergoes three stages of regeneration—inflammation (localized swelling), new tissue formation (rebuilding), and maturation (remodeling), resulting in healed wound areas that are generally weaker than uninjured skin. These wound healing stages are not only complex but also fragile, and it is well known that wound healing kinetics such as speed and efficiency vary in each individual depending on stress level, age, sex, and lifestyle [2]. Additionally, pathological conditions can interfere with the normal wound healing process and may lead to impaired or delayed wound healing such as diabetic ulcers and chronic wounds [3]. In turn, excessive healing can also lead to the formation of non-functional fibrotic tissue and impaired vascularization [3]. The investigation of the cellular and molecular mechanisms of impaired cell migration and wound closure is necessary to understand how to improve overall healing dynamics. Using predictive wound healing models for different skin pathologies [4] is consequently vital in devising novel therapeutic strategies to elevate patient suffering, reduce scaring, and eliminate chronic wounds. As wound healing is guided by fine-tuned molecular processes, both the effect and the dosage of therapeutic agents need to be investigated for cutaneous cells. To study tissue regeneration processes and screening treatment options, various in vitro wound healing assays have been established to elucidate the most influential factors and mechanisms that govern cell proliferation and migration. Here, cell-free areas (wounds) within cell layers are induced using mechanical (scratching), thermal, laser as well as electrical methods [5]. Among these, the scratch assay is still the most widely used in vitro wound healing assay where cells are manual removed from a cell layer using pipet tips. To overcome the many shortcomings of conventional in vitro wound healing assays including reproducibility, manual labor, and flexibility, a number of microfluidic wound-healing assays have been developed in recent years to provide automated, miniaturized, and integrated cell analysis platforms.

Consequently, this review focuses on the current state of established microfluidic wound healing assays that assess the role of cell migration in the overall wound healing process including methods for wound generation, wound healing analysis schemes, and the influence of molecular stimuli and/or inhibitors (e.g., oxygen, serum content, growth factors, and small molecules) in mechanistic studies on cell migration and wound healing. 

## 2. A Brief Overview of Wound Healing: Process, Cells, and Pathways 

**Figure 1:**
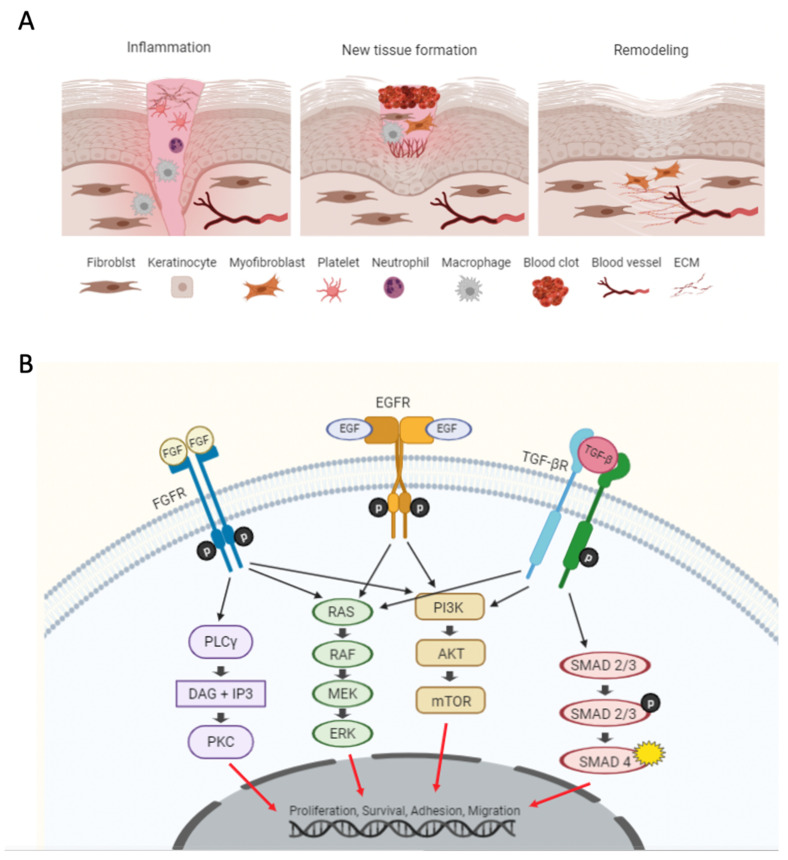
(**A**) Overview of the cellular processes during the three wound healing stages. (**B**) Schematic representation of pathways involved in wound healing, including receptors for fibroblast growth factors (FGFs), epidermal growth factors (EGFs), and transforming growth factor β (TGF-β).

A series of activated intracellular and intercellular pathways initiate the wound healing and repair process, as shown in Figure 1A [6], comprising three distinct stages: (1) inflammation, (2) new tissue formation, and (3) tissue remodeling [1,3,7,8]. Malfunction in one or more of these stages can result in abnormal or defective wound healing, such as excessive cell proliferation (e.g., keloid scars), a lack of wound closure (e.g., diabetic ulcers), and chronic wounds [1,3,9]. It is also important to highlight that various cell types such as keratinocytes, fibroblasts, endothelial, and immune cells are intricately involved in cell proliferation, differentiation, and migration during wound healing stages [3,10]. 

The first stage of wound healing is inflammation, which occurs as an immediate response to a break in the epithelial barrier. In this stage, inflammatory pathways prevent further bleeding and infection [3]. Neutrophils, macrophages, and monocytes migrate into the wound site to clean the wound region from pathogens and dead cell debris. The crosslinking of the extracellular matrix (ECM) and fibrin forms a scaffold for platelet plug formation. Secreted mediators from platelets attract fibroblasts and white blood cells into the wound site [10]. Neutrophils clean the wound area and produce pro-inflammatory cytokines such as interferon-gamma (IFN-γ) and interleukin 1 beta (IL-1 β) [3,9]. In turn, these cytokines lead to the expression of adhesion factors such as selectins. Once monocytes have migrated into the wound site, they readily differentiate into active macrophages responsible for the phagocytosis of pathogens and matrix debris. These infiltrating cells also express inflammatory cytokines and growth factors, such as platelet-derived growth factor (PDGF), transforming growth factor β (TGF-β), and endothelial growth factor (VEGF), necessary for fibroblast cell proliferation and de novo tissue formation [7,10]. 

In the second stage of wound healing, known as the proliferative stage, re-epithelialization and vascularization occurs to fill and cover the wound [10]. The migration, proliferation, and maturation of keratinocytes and dermal fibroblasts lead to the de novo formation of tissue known as granulation tissue [11]. Here, a fraction of these fibroblasts further differentiate into myofibroblasts [3], which contribute to wound contraction by expressing alpha-smooth muscle actin (α-SMA) [6,8,10]. Additionally, the synthesized ECM (consisting mainly of collagen type I and III [6]), which is produced by fibroblasts and myofibroblasts, is also necessary to attract cell ingrowth and initiate wound closure. VEGF, secreted by epidermal cells, further stimulates capillary sprouting and neovascularization in the wound bed to ensure sufficient oxygen and nutrient supply [1]. However, the most critical biochemical regulators in this stage are fibroblast growth factor 2 (bFGF) [1,3], VEGF, and TGF-α, which regulate angiogenesis, wound closure, granulation tissue formation, and re-epithelialization [12]. 

In the remodeling stage, anti-inflammatory cytokines such as interleukin 10 (IL-10) start to regulate immune cell infiltration (i.e., macrophages), as well as collagen type I synthesis, through the regulation of cell proliferation and ECM remodeling [7,10]. After wound closure is accomplished, the ECM composition changes and thicker collagen fibers start to form to increase tissue resilience. In this stage, the remaining fibroblasts, macrophages, and endothelial cells undergo apoptosis and are removed from the tissue [1], while myofibroblasts continue producing ECM during the fibrosis process and wound contraction. The alignment of fibers in one direction and the transformation of the granulation tissue eventually result in the formation of scar tissue, which in known to exhibit fewer cells and is less vascularized than healthy tissue [3].

It is important to note that dermal fibroblasts play a vital role in all stages of cutaneous wound healing and connective tissue regeneration [13], and they are therefore frequently used as representative in vitro models in preclinical and clinical studies [14]. For instance, it was demonstrated that the phenotypical changes of fibroblasts removed from chronic wounds exhibit an altered cytokine release pattern and decreased cell motility [15,16]. Additionally, fibroblast cocultivation with keratinocytes can further stimulate cutaneous basement membrane formation, resulting in a more physiological matrix architecture [17]. Consequently, in vitro 3D co-culture systems containing dermal fibroblasts and keratinocytes have been extensively used as a skin equivalents for wound healing studies and drug-screening applications [17]. Furthermore, fibroblast cell migration into the wound site is known to activate signaling cascades such as ERK, MAPK, and TGF-β, thus producing bFGF and TGF-β [3]—all important pathways in the process of wound closure and physiological wound healing. The TGF-β/Smad pathway particularly plays an essential role in tissue homeostasis under normal conditions because it suppresses keratinocyte proliferation [3]. In more detail, TGF-β receptors phosphorylate Smad2 and Smad3, and they form a complex with Smad4. The Smad4 complex is translocated into the nucleus, where it regulates gene expression for inflammation, cell proliferation, matrix synthesis, and cell migration. Furthermore, integrin gene expression promotes keratinocyte migration into the wound site [6,18], where the integrin-induced TGF-β secretion further promotes the locomotion of myofibroblasts, which are vital players in wound contraction and healing. For instance, researchers have shown that an artificial increase in the TGF-β level leads to more directional changes in migrating fibroblasts and reduces scar formation [18]. In keloid scars, a more serious form of excessive scarring, as well as the upregulation of TGF-β and IL-1, has been shown to dysregulate collagen synthesis towards unwarranted fibrosis, thus resulting in large, tumorous neoplasm [19]. Another important regulatory pathway is the MAPK pathway, which is involved in the regulation of cell proliferation and differentiation events. In addition to the activation of the ERK, MAPK, and TGF-β signaling cascades, other pathways—like the AKT pathway that mainly regulates cell survival and the PLCγ pathway that guides cell morphology, migration, and adhesion—are involved in the healing process [20]. The activation of EGFR leads to the phosphorylation of downstream proteins and the activation of signaling pathways such as PI3K/AKT and MAPK [3], which stimulates re-epithelization by promoting keratinocyte proliferation and migration [7].

In summary, the complex signaling cascades and pathway activations that take place during the wound healing process govern the cell-to-cell and cell-to-matrix interactions that lead to wound clearance, rebuilding, and maturation. The failure to progress in any of the three stages of wound healing can therefore lead to impaired healing, chronic wounds, and excessive scar formation. 

## 3. Conventional In Vitro Wound Healing Assays 

**Figure 2:**
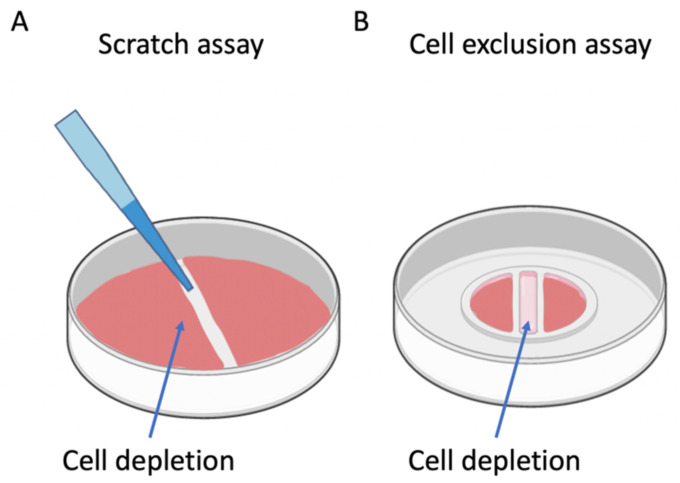
In vitro wound healing assays. (**A**) Scratch assays most frequently use pipette tips to manually scratch and remove cells from a cell monolayer. (**B**) Cell exclusion assays block a wound area before cell adhesion with a physical barrier insert removed after the establishment of the cell layer integrity to create a well-defined wound.

The ability of mammalian cells to migrate represents a fundamental aspect in biology that is essential for embryonic development, immune response, cancer metastasis, and wound healing [21]. Therefore, cell migration analysis has become a valuable and indispensable tool to study the mechanisms underlying cell motility and the effects of stimulants on cell migration [22]. Though there are a number of in vivo animal models, such as excision [23], incision [24], and burn animal models, for studying wound healing processes at the whole organism level [25], the majority of cell migration analysis are performed using in vitro wound healing assays. Figure 2A,B shows a schematic overview of the two most commonly used in vitro cell migration and wound healing assays including scratch and cell exclusion methods for cell migration analysis [21]. The main applications of in vitro wound assays are (1) analyzing collective cell migration, (2) analyzing skin cell migration for cutaneous wound closure studies, (3) discovering the effects of ECM on cell migration, (4) studying the mechanism of cancer metastasis, and (5) screening for drugs [21,26]. The basic principle behind all in vitro wound healing assays is to either exclude or remove a portion of a cell monolayer using mechanical, enzymatic, or thermal methods to create cell-free areas [22]. Here, cell culture conditions, cell seeding density, and wound size are the main parameters that can affect the reproducibility of in vitro wound healing assay [22]. 

A scratch assay is still the most common method used for cell migration assessment, despite its many shortcomings such as a lack of standardization, high variability, and low reproducibility. Scratch assays are widely used in different research fields such as fundamental biology, drug screening, cancer metastasis, immunology, and wound healing [21]. The main tasks of this 2D assay are (1) the preparation of a cell monolayer in culture, (2) the scratching of the monolayer to create a cell-free area, and (3) microscopy and imaging [22]. Visual cell migration analysis provides information on vital biological performance parameters, including cell migration speed and overall wound closure rate [21]. Scratch assays mimic mechanical injuries due to the damage they cause to cells, leading to the release of growth factors and cytokines [21]. However, the release of cellular contents from damaged cells at the wound edge can interfere with migration processes [21,27]. Additionally, manual wounding by various scratching pressure and angles [22] causes variations in wound size and quality, consequently limiting its feasibility for high-throughput screening applications [27,28]. Nevertheless, due to their simplicity and low costs, manual scratch assays are still the method of choice [29,30,31] to study pathological wounds and the regulatory effect of growth factors on cell migration [32]. An alternative method of creating cell-free areas is to exclude cells using silicone inserts [33] and stoppers to prevent cell growth in defined areas. After cell adhesion and cell growth, the culture inserts are removed to monitor cell migration and wound closure [21]. This method’s advantage is generating more reproducible wound sizes; however, inserts are more expensive, and the exclusion of cells cannot mimic the mechanical process of traumatic wound process. The improper adhesion of the inserts into the substrate can also lead to cell ingrowth into the cell-free gap [22]. 

In summary, conventional migration and wound healing assays based on cell exclusion or removal feature a range of limitations and are associated with endpoint detection, have non-linear or uncontrolled gradients, lack reproducibility, are not automation-friendly, require the manual removal of inserts, damage matrix coatings, and have variability between control and experiment scratching. Consequently, next generation wound healing assays need to address variability, show flexibility in application, permit live cell imaging, perform high-content analysis, and feature simple on step protocol (automation-friendly). 

## 4. Advanced Microfluidic Wound-Healing Assays

To address the above-outlined shortcomings, various microfluidics and lab-on-a-chip systems have been developed to improve standard wound healing assays with various potential applications such as drug discovery, diagnostics, and basic research. The general principle of microfluidic chip technology is to create a platform for miniaturized and automated bioassays [34]. The small volumes required in miniaturized microfluidic devices allow for scalable, high-throughput assays for cell-based analysis [27]. These technologies can be used as state-of-the-art personalized devices, particularly for diagnostics and drug screening because of well-established plastic mass production technology [35]. The high costs of preclinical testing, time-consuming research, and time-consuming development are the main challenges in the pharmaceutical industry. The failure of drug efficacy and effectiveness during a clinical trial due to a lack of valid preclinical results can be financially disruptive and cause harm to human patients [34]. Preclinical experiments with animal models, in many cases, fail to mimic human body responses to specific treatments. Moreover, the response to treatment can vary from patient to patient due to genetic and lifestyle differences. Therefore, using an intermediate in vitro model with human cells can provide more realistic predictions in the early stages of drug testing and can prevent high costs [27,36]. The fabrication of disposable and affordable microdevices creates a platform for parallel and high-throughput analysis. Commonly, silicone-based polymers are preferred for the fabrication of microdevices due to good biocompatibility. Moreover, the fabrication of fluid channels using molding, hot embossing, and cutting is known to be relatively straightforward [34]. Polydimethylsiloxane (PDMS), a synthetic silicone-based polymer, is widely used for microdevice fabrication. The optical transparency and gas permeability for CO2 and O2 diffusion of this material make it ideal for cell-culture purposes [37]. The transparency of the microdevice system makes the microscopy and tracking of the fluid and cells possible. Other materials and devices such as glass silicon or metal with integrated sensors can be applied to a microfluidic device, depending on its diagnostic or screening use [34].

**Figure 3:**
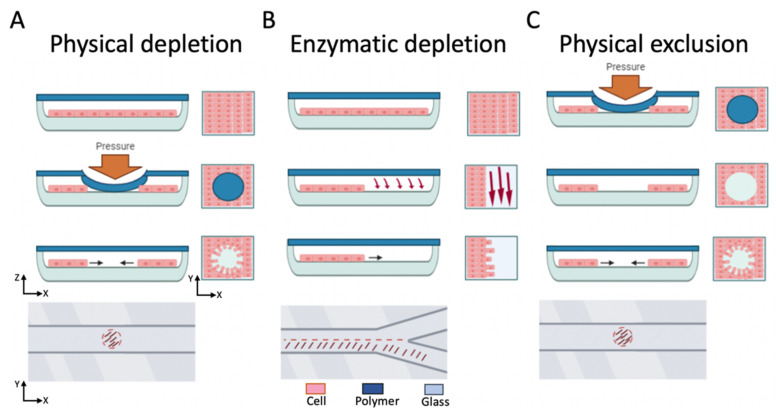
Overview of state-of-the-art microfluidic wound healing assays, including cell-depletion (physical or enzymatic) and physical cell exclusion approaches in microfluidic channels. (**A**) the mechanical cell depletion approach, (**B**) the enzymatic cell depletion approach, and (**C**) the cell exclusion approach. In each approach cross-section, and top views of the microchannel during and after wounding are illustrated.

Over the last two decades, many on-chip wound healing assays based on microfluidics have been reported using various microchannel designs that create cell-free areas by either cell exclusion or cell depletion [38], as shown in Figure 3. For cell depletion, thermal, electric, enzymatic, or mechanical principles remove cell portions from confluent monolayers to result in cell-free wound areas. In contrast, microfluidic cell exclusion assays initially block cell adhesion on parts of the substrate with an actuated structure or removable cover before cell seeding. The removal of the cell blocking structure after cell attachment creates a wound defect. Cell migration assays for the analysis of molecular processes in wound healing while using microdevices to analyze cell–cell interactions [38], skin inflammation models-on-chip [39] (including hydrogel cell migration assays [40]), and chemotaxis chips [41] cannot be regarded as wound healing assays per se and are not elaborated upon in more detail in the current review.

### 4.1. Exclusion 

**Figure 4:**
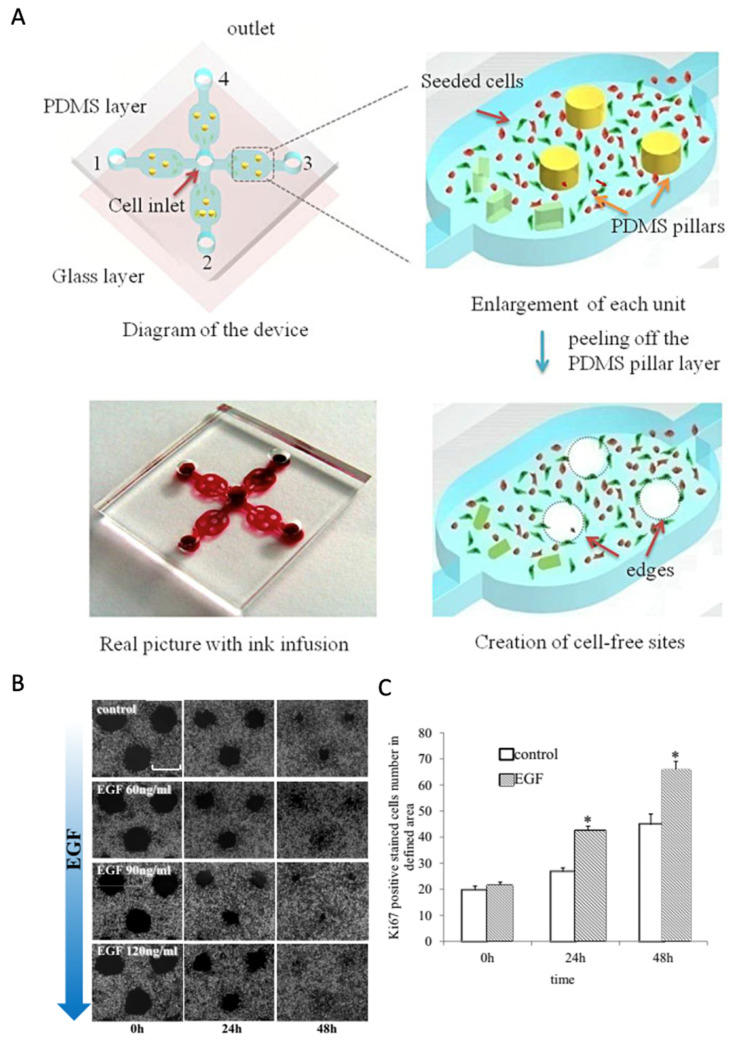
Microfluidic wound healing assays based on physical cell exclusion. (**A**–**C**) Pillar-based microfluidic wound healing to analyze the influence of (**B**) EGF concentration on wound closure and (**C**) the number of proliferative cells. (* *p* < 0.05 vs. control). Adapted with permission from ref. [28]. 2021, Elsevier.

Instead, the remaining sections focus on how chip-based assays (Table 1) improve biological insights into cellular and molecular wound-healing processes and drug-screening studies. For instance, Zhang et al. used microfluidic technology to establish an in vitro wound-healing assay based on the exclusion method for creating wound areas based on pillar structures [28]. Using the PDMS pillar approach shown in Figure 4A–C for human gastric epithelial GES-1 cells, the authors investigated the stimulatory effects of EGF with a 50% increase in cell proliferation and a concentration-dependent increase in cell migration speed. Poujade et al. used micro stencil cell exclusion to characterize focal adhesion quality on various substrates (e.g., cell-culture plastic and fibronectin-coated glass) and the overall impact of bio-interface properties on wound closure speed [42]. Gao et al. presented a wound-healing assay based on multi-layered microfluidics [27] to create a cell-free area by applying mechanical force between two microchannels. Consecutive pressure release allowed for cell migration into the cell-free areas. This exclusion approach enhanced the inhibition of melanoma cell migration over 18 h, using very few numbers of EGFR+ and BRAFV600E wild-type MV3 cells. Even though the authors presented a feasible tool for personalized wound healing applications requiring low cell numbers, the study itself was on cancer biology. 

### 4.2. Enzymatic Depletion 

**Figure 5:**
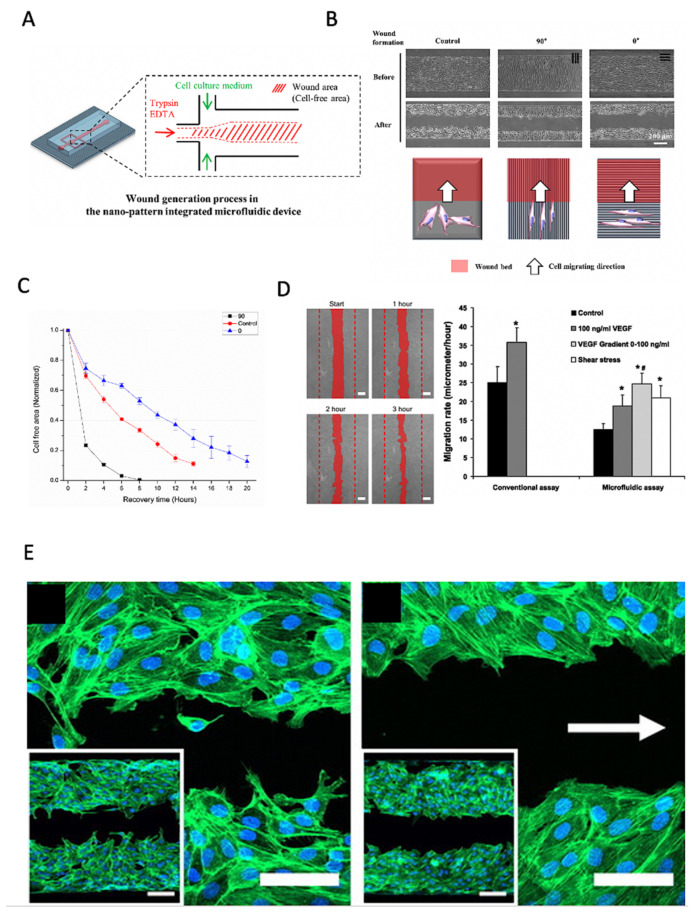
Enzymatic microfluidic wound healing assays based on the laminar flow patterning of fluids. (**A**–**C**) Influence of the on-chip nanopattern direction on wound healing speed using enzymatic depletion of a central cell-free area using trypsin. Adapted with permission from [48]. (**D**,**E**) Influence of flow direction, shear, and VEGF on (**E**) endothelial migration rate and (**D**) wound healing directionality. (* Significant increase compared with control values (Student’s *t*-test, *p* < 0.05). # Significant increase compared with 100 ng/mL VEGF165 treatment (Student’s *t*-test, *p* < 0.05). Adapted with permission from ref. [49]. 2021, Elsevier.

In addition to mechanical depletion or exclusion methods, enzymatic detachment is another frequently used wounding method based on the depletion of cell-free areas by streaming enzymatic detachment solution over portions of confluent cell monolayers (see Figure 5). 

Nie et al. reported an NIH-3T3 fibroblast cell migration assay using enzymatic wound creation by the hydrostatic laminar flow patterning of trypsin/EDTA on one side of the microfluidic channels [43]. Similarly, Lin et al. investigated the influence of shear and wound size on cell migration and the wound closure speed of NIH-3T3 fibroblasts using a polymethylmethacrylate (PMMA)-based microdevice and showed that increased flow rate better accelerated wound healing in 6-mm-wide wound defects than in 3-mm-wide wound defects [44]. However, fibroblasts exposed to higher shear forces before wounding in 3 mm channels showed slower migration speeds. Similarly, Conant et al. analyzed the proliferation and migration speed of rat epithelial cells under starvation conditions [45]. Wie et al. investigated the effects of microchannel height, surface coating, and chemokine stimulation on the cell migration rate of primary human vascular smooth muscle cells (VSMCs) to confirm that FBS, PDGF, TNF-α, and chamber height positively promote cell migration speed [46]. Conde et al. used a single-channel microdevice with three inlets to enzymatically induce a central wound area with two opposing wound edges on melanoma cell monolayers [47], while Lee et al. performed a similar wound healing assay, as shown in Figure 5A–C [48], on NIH-3T3 fibroblasts to investigate how the direction of surface nanopatterns could accelerate wound closure.

Similarly, van der Meer et al. used an enzymatic wound-healing chip for HUVEC endothelial monolayers to show that VEGF gradients, as well as fluid shear, improve endothelial cell migration speed. However, shear severely impacts the directionality of migration along the fluid flow direction (see Figure 5D,E) [46]. Furthermore, Murrel et al. analyzed how cell spreading and motion are influenced by enzymatic cell depletion at the leading edge of tight epithelial cell layers, and they concluded that reactive oxygen species generation plays a vital role in cell migration inhibition [50]. Jeong et al. presented a migration assay for endothelial cell migration and sprouting using microfluidic chip technology [51], providing endothelial cells with a 3D microenvironment in two scaffold channels to investigate the effect of growth factors on cell migration behavior. Shih et al. advanced a conventional enzymatic endothelial wounding assay with an on-chip chemical oxygen concentration generator to demonstrate that the influence of oxygen gradients is more severe than homogenous hypoxic oxygen tension on the directionality of endothelial cell migration towards low oxygen concentrations [52]. Only the gradient, but not stable hypoxia, induced this migratory directionality, which was not affected by migration inhibitory drugs.

### 4.3. Physical Depletion

**Figure 6:**
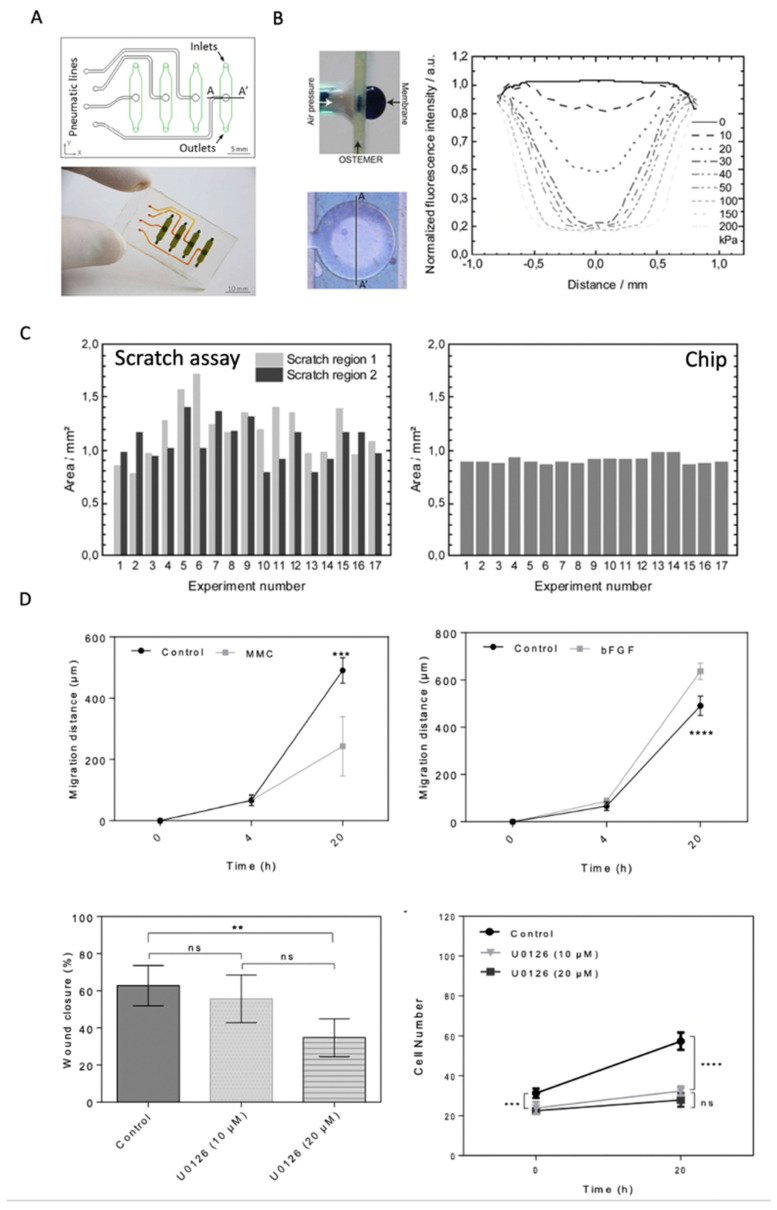
(**A**) Wound-healing lab-on-a-chip system with four individual pneumatic and fluidic cell chambers. (**B**) Pneumatic actuation of a flexible membrane within the microfluidic device. (**C**) Direct comparison of a conventional scratch assay’s reproducibility and precision compared to pneumatically-actuated, automated physical cell depletion method for endothelial cells (HUVECs). Adapted from [53] with permission of The Royal Society of Chemistry. (**D**) Effect of growth factor bFGF and inhibitory agents mitomycin C (MMC) and MEK-inhibitor U0126 on dermal fibroblast migration and proliferation dynamics. (ns, non-significant; ** *p*  <  0.01; *** *p*  <  0.001; **** *p*  <  0.0001). Adapted with permission from ref. [54]. 2021, Elsevier.

Like the actuated pillar approaches mentioned earlier, Sticker et al. developed two microdevices for performing automated cell migration assays based on both the cell exclusion and cell depletion methods for wound creation (see Figure 6A–C) [53]. Both devices contained three layers, with the difference in the middle layer. The cell depletion device comprised a top pneumatic layer, a middle PDMS layer, and a bottom layer with multiple microchannels. This technology platform created highly automated and reproducible wounds for both methods to show how TNF-α and mitomycin C decreased wound healing speed. A recent follow-up study by Shabestani Monfared et al. [54] adapted this approach using PDMS rapid prototyping by xurography to automatically create more wounds with a single pneumatic actuation cycle. 

The authors applied their microsystem to investigate the effect of medium supplements such as growth factors and proliferation inhibitory drugs on human dermal fibroblast (HDF) cell migration (see Figure 6D). For instance, fibroblast stimulation with bFGF increased fibroblast wound closure while increasing migration distance, whereas Mitomycin C decreased the cell migration rate due to proliferation inhibition. The MEK inhibitor U0126 showed no effect on migration speed and total wound closure relative to the untreated control samples by selectively inhibiting only cell proliferation by approximately 32%. With RSD around 3%, both mechanical studies outperformed the standard deviation of conventional scratch assays independently of cell type (e.g., endothelial or fibroblast cells) and wound diameter (e.g., 1 vs. 1.5 mm^2^), highlighting how one can improve the reproducibility and comparability of wound-depletion approaches.

Wound healing assays using microfluidic technologies are based on cell migration into cell-free areas created using various physical and biochemical methods. The most common strategy to create cell-free areas (wounds) in cell monolayers is enzymatic detachment using laminar flow patterning, which influences the cell dynamics on the edges due to the enzyme interaction with cells. Mechanical approaches, however, present more realistic strategies for on-chip wounding. However, these methods often require a more complex microdevice design regarding the number of layers and channels and additional controllers of actuators that deplete or exclude cells. On the one hand, laminar flow patterning is most often used and requires syringe pumps, while hydrostatic fluid handling can obviate pumps and improves scalability for drug-screening applications. On the other hand, applying pressure via pneumatic actuators requires pressure lines and pressure controllers to automate the wounding process via cell exclusion or cell depletion. Once the wounding strategy is selected, the challenge is using these microdevice solutions for the biological read-out of wound healing processes. Microfluidic systems are used for wounding to frequently investigate either human endothelial cells (HUVECs) or mouse embryonic fibroblasts (3T3 fibroblasts). The selection of HUVECs is relevant for the investigation of microlesions and ruptures that occur throughout the human vascular system because they are well-established, easy to handle, and commercially available. Embryonic fibroblasts are a questionable choice as a relevant model for dermal wound healing processes that can be predictive for human wounds and should be considered for very early stage and proof-of-principle studies. Keratinocytes, combined with dermal human fibroblast cells and other more volatile cell populations (e.g., monocytes), would be the best option for wound models capable of predicting the complex processes of wound healing in general. These cells can be eventually integrated into state-of-the-art, commercial, full-thickness dermal and epidermal skin models for wound healing assessment. In line with the embryonic tissue problem, cancer cell lines are frequently used for cell migration and wound healing studies even though these models are more relevant for research fields such as cancer cell migration processes in metastasis, such as tumor cell extravasation and invasion. Microfluidic technologies have outperformed conventional wound healing assay because of the precise control of vital microenvironmental culture parameters, including oxygen and fluid shear. The automation of on-chip wound healing has further improved the reproducibility of wound shape and size by eliminating human errors during the wounding process. Finally, the miniaturization of wound healing assays using chip technology has the main advantage of using smaller amounts of reagent and cell materials, which is an essential aspect for the scalability of pharmaceutical wound healing investigation for personalized medicine in preclinical research fields such as a chronic wound healing.

## 5. Outlook

In order to use microfluidic wound-healing assays as state-of-the-art tools for wound healing research, 3D tissue models using keratinocytes, fibroblasts, endothelial, and immune cells should be further advanced to better recapitulate anatomical and pathophysiological processes at the cellular and molecular levels. Such complex wound models would have the capacity to be used as skin disease models for research on and the drug-screening of pathologies including diabetic wounds and skin fibrosis. The establishment of full-thickness pathological wound models would provide a more scalable and cost-efficient technology platform for drug screening and medicine development when combined with automated and quantitative analysis schemes (e.g., automated sampling, integration into automated analysis routines, and the integration of microsensors). Combing wound healing microdevices with dynamic cultures by including patient-derived cells/stem cells would lead to the development of personalized medicine based on a patient’s unique genetic background. This approach could significantly reduce the need for animal testing and could be used to develop patient-specific devices for drug screening or cosmetic testing due to the small number of cells and drugs needed for each wound healing assay.

## Figures and Tables

**Table 1:** Assay types, material of devices, and cell types for wound healing assessment.

Assay Type	Microdevice Material	Cell Types	Ref.
Cell Exclusion	PDMS and glass	Gastric epithelial GES-1 cells	[28]
	PDMS, glass, and cell-culture plastic	Epithelial cells	[42]
	PDMS	Human melanoma cells	[27]
Enzymatic cell depletion	PDMS and polystyrene	NIH-3T3 fibroblasts	[43]
	PMMA	NIH-3T3 fibroblasts	[44]
	PDMS and cell-culture plastic	Rat epithelial cells	[45]
	PDMS and glass	VSMCs	[46]
	PMMA	Human melanoma cells	[47]
	PDMS and PUA	NIH-3T3 fibroblasts	[48]
	PDMS and glass	HUVECs	[49]
	PDMS	Moues epithelial Cells	[50]
	PDMS	HUVECs	[51]
	PDMS	HUVECs	[52]
Physical cell depletion	PDMS and glass	HUVECs	[53]
	PDMS and glass	HDFs	[54]

## Data Availability

Data are available under reasonable email request.
